# Genomic characterisation and dissection of the onset of resistance to acetyl CoA carboxylase-inhibiting herbicides in a large collection of *Digitaria insularis* from Brazil

**DOI:** 10.3389/fgene.2024.1340852

**Published:** 2024-02-19

**Authors:** Deepmala Sehgal, Claudia Oliveira, Sandra Mathioni, Stephanie Widdison, Will Plumb, Breno Campos, Shiv Shankhar Kaundun

**Affiliations:** ^1^ Syngenta Ltd., Jealott’s Hill International Research Centre, Bracknell, United Kingdom; ^2^ Syngenta Crop Protection, Holambra Research and Development Center, São Paulo, Brazil

**Keywords:** *Digitaria insularis*, genotyping-by-sequencing, genetic diversity, GWAS, genomic prediction, ACCase inhibitors

## Abstract

An in-depth genotypic characterisation of a diverse collection of *Digitaria insularis* was undertaken to explore the neutral genetic variation across the natural expansion range of this weed species in Brazil. With the exception of Minas Gerais, populations from all other states showed high estimates of expected heterozygosity (H_E_ > 0.60) and genetic diversity. There was a lack of population structure based on geographic origin and a low population differentiation between populations across the landscape as evidenced by average Fst value of 0.02. On combining haloxyfop [acetyl CoA carboxylase (ACCase)-inhibiting herbicide] efficacy data with neutral genetic variation, we found evidence of presence of two scenarios of resistance evolution in this weed species. Whilst populations originating from north-eastern region demonstrated an active role of gene flow, populations from the mid-western region displayed multiple, independent resistance evolution as the major evolutionary mechanism. A target-site mutation (Trp2027Cys) in the ACCase gene, observed in less than 1% of resistant populations, could not explain the reduced sensitivity of 15% of the populations to haloxyfop. The genetic architecture of resistance to ACCase-inhibiting herbicides was dissected using a genome wide association study (GWAS) approach. GWAS revealed association of three SNPs with reduced sensitivity to haloxyfop and clethodim. *In silico* analysis of these SNPs revealed important non-target site genes belonging to families involved in herbicide detoxification, including UDPGT91C1 and GT2, and genes involved in vacuolar sequestration-based degradation pathway. Exploration of five genomic prediction models revealed that the highest prediction power (≥0.80) was achieved with the models Bayes A and RKHS, incorporating SNPs with additive effects and epistatic interactions, respectively.

## Introduction

*Digitaria insularis*, commonly known as sourgrass, is an outcrossing perennial grass weed native to Central and South America, where glyphosate-tolerant corn and soybean varieties are primarily grown as a double-crop year system. The extensive glyphosate-based weed management in South America has led to widespread evolution of glyphosate resistant populations of *D. insularis* over the years (Heap, 1999). In Brazil, glyphosate resistant sourgrass populations were first reported from soybean and maize fields in 2008 (de Carvalho et al., 2011). In just over a decade, *D. insularis* has become one of the most persistent weed species competing with soybean, corn and cotton. In soybean, yield losses to sourgrass infestation can reach up to 80% and it is a major concern to the agricultural sector (Lopez Ovejero et al., 2017; Gazziero et al., 2019). *D. insularis* propagates by seed and asexually by rhizomes, which makes it even more challenging to control in the field as compared to many other weeds.

Acetyl coenzyme A carboxylase (ACCase) inhibiting herbicides have been used intensively for post-emergence control of *D. insularis* in Brazil, which substantially increased selection pressure for this class of herbicides. A total of 49 weeds have already evolved resistance to ACCase inhibitors, conferred predominantly by target-site resistance (TSR) mechanism. The TSR mechanism involves point mutation(s) in genes encoding the protein targets of herbicides affecting the binding of the herbicide at or near catalytic domains. Several mutations at ACCase codons Ile1781, Trp1999, Trp2027, Ile2041, Asp2078, Cys2088 and Gly2096 in the carboxyltransferase (CT) domain of the enzyme have been associated with resistance to ACCase-inhibiting herbicides (Powles and Yu, 2010; Beckie and Tardif, 2012; Kaundun, 2014; Takano et al., 2020). The non-target site resistance (NTSR) mechanisms are more complex and can include one or more physiological processes resulting in reduced absorption and translocation of the herbicides and/or their enhanced metabolism or sequestration. The metabolism-based NTSR mechanism, involving the increased activity of enzymes such as cytochrome P450s, glutathione S-transferases and/or Uridine 5′-diphospho (UDP)-glucosyl transferases, has been reported for ACCase inhibitors in a few grass weed species (Powles and Yu, 2010; Yu et al., 2013; Han et al., 2016; Iwakami et al., 2019).

Hitherto, limited knowledge exists on the genetic diversity and population genetic structure in *D. insularis* as is the case with most other weed species (Gonçalves Netto et al., 2021). The information on the genetic diversity and population structure in *D. insularis* is important to increase our understanding of the genetic structure and gene flow across natural expansion area of this weed species. Recent advances in next-generation sequencing (NGS) technologies and bioinformatic and statistical tools have opened new vistas to characterize plant genomes at a much greater depth than before. Genotyping by sequencing (GBS), particularly, has been used extensively in crops to assess genetic diversity, investigate population genetic structure and gene discovery for a plethora of traits using genome wide association study (GWAS) approach (Kim et al., 2016; Sehgal et al., 2017; 2020; Shi et al., 2020; Abou-Khater et al., 2022). It is a rapid, high-throughput, and cost-effective method for the simultaneous discovery of single nucleotide polymorphisms (SNPs) and genotyping of target germplasm (Poland and Rife, 2012). The GBS technology has made it possible to generate thousands to millions of SNPs with a high resolution with and without the availability of a reference genome in weed species (Küpper et al., 2018).

The present study aims to i) characterize the genetic diversity in a large Brazilian collection of *D. insularis* using the GBS approach ii) determine the population genetic structure and investigate pattern of gene flow between populations iii) identify marker trait associations (MTA) for resistance to reduced sensitivity to ACCase inhibitors such as haloxyfop and clethodim using the GWAS approach and iv) investigate genomic prediction (GP) models to explore the potential of this approach for predicting NTSR. The present study is the first report of a detailed genotypic characterization of the largest collection of *D. insularis* and a preliminary investigation to explore the potential of GWAS approach and GP models dissecting NTSR in its natural diverse populations.

## Materials and methods

### Plant material

A total of 205 *D. insularis* weed populations were used in the study. The populations were collected from the most relevant soybean- (and corn) growing regions across Brazil inhabiting crop fields and crop margins in the southern, mid-western, south-eastern and north-eastern regions. Briefly, these 205 populations originated from seven states including Mato Grosso (77), Mato Grosso do Sul (10), Minas Gerais (19), Bahia (24), Goiás (20), Paraná (PR) and Rio Grande do Sul (18). The detail information on the geographic origin and geographic coordinates are provided in the (Supplementary Table S1). Some collection sites (1/5th of the collection) had a history of multiple herbicide treatments on the crop including glyphosate, ACCase inhibitors such as haloxyfop or clethodim alone or in mixtures and/or ALS inhibitors (Supplementary Table S1). The populations were collected in 2020 and 2021 and were investigated for their sensitivity to ACCase inhibitors haloxyfop and clethodim at multiple dose rates by Weed Research team at Brazil Resistance Management Laboratory in Uberlândia, Brazil, as part of resistance monitoring studies.

### Herbicide resistance screening of *Digitaria insularis* collection

The rates used in the population screening were initially determined in a pilot study using ten *D. insularis* sensitive populations sampled in Brazil urban areas with no history of herbicide application. Informative herbicide rates were determined as 7.8; 15.0, 27.0 and 64.0 g a. i ha^-1^ for haloxyfop and 27.0, 54.0 and 108.0 g a. i ha^-1^ for clethodim.

Seeds from all *D. insularis* populations were germinated and planted in 1 L pots filled with a commercial substrate to produce around 15 plants per pot. Each combination of population and herbicide dose was replicated 3 times (45 plants tested per treatment) in a completely randomized design. The herbicide treatments were sprayed on plants at the 4 leaf-stage, in a spray chamber equipped with flat fan nozzles calibrated to deliver 200 L ha-1 at 200 kPa pressure. Plant control was evaluated 21 days after treatment, using a visual scale of 0%–100%; 0% represents healthy plants and the absence of symptoms, and 100% represents the death of the plant.

### DNA extraction, genotyping-by-sequencing (GBS) library preparation and sequencing

Four pinches of seeds were sown in a punnet of size 18 cm × 6 cm filled with peat keeping 3 cm between pinches. For each population, two such punnets were sown. The punnets were watered manually and put on trolleys in a glass house with controlled conditions (day temperature, 24°C; night temperature, 18°C; light, 16 h; humidity −65%). The punnets were watered daily for 3 weeks. After 3 weeks, a 1 cm × 2 cm of leaf sample was cut from 25 individual plants for each population and pooled into a 14 mL falcon tube. The falcon tubes were stored in a −80°C freezer until further manipulation.

The leaf samples were dried in a freeze dryer for three consecutive days and nights by keeping the shelves at a contact temperature of 1.0°C and freezer at −60°C. After freeze drying, the samples were shipped to LGC Genomics GmbH, Germany for DNA extraction, reduced representation library preparation and sequencing.

Genomic DNA extraction was performed from the pooled samples using the sbeadex™ maxi plant kit (LGC) on KingFisher Flex (after lysis step) followed by a spectrophotometric quantification step using Nano Drop 8,000 (Thermo Fisher Scientific). Reduced representation library preparation was done by the standardized ddRAD protocol at LGC Genomics. Briefly, 100 ng of genomic DNA were digested with 2 units each of *Apek* I and *Pst* I enzymes (NEB) in 1 times NEB buffer 3.1 in 20 μL volume for 1 hour at 37°C. The restriction enzymes were heat inactivated by incubation at 75°C for 60 min. The detailed protocol for the ligation reaction, library purification, amplification and normalization were performed according to the standardized ddRAD protocol at LGC Genomics, GmbH. The library was size selected on a LMP-Agarose gel, removing fragments smaller than 300 bp and those larger than 500 bp. Sequencing was done on an Illumina NovaSeq 6,000 (150bp paired-end read).

### Genotypes and SNP filtering

Demultiplexing of all libraries for each sequencing lane was done using the Illumina bcl2fastq v2.20 software. Demultiplexing of library groups into samples was done according to their inline barcodes and verification of restriction site. No mismatches or Ns were allowed in the inline barcodes, but Ns were allowed in the restriction site. Reads with final length <20 bases were rejected and reads with 5′ ends not matching the restriction enzyme site were also discarded. The reads were quality trimmed at 3′-end to get a minimum average Phred quality score of 20 over a window of ten bases. The mapping of quality trimmed reads on the *D. insularis* reference genome v01.0 (available at Weedpedia, https://weedpedia.weedgenomics.org/) was done using BWA-MEM v0.7.12. One combined alignment file of all samples in the BAM format was used for variant discovery and genotyping of samples with Freebayes v1.2.0. Filtering of variants was done using the following GBS-specific rule set;

1. The read count for a locus must exceed 8 reads
2. Genotypes must have been observed in at least 66% of samples
3. Minimum allele frequency across all samples must exceed 5%.

### Genetic diversity and population differentiation

The genetic diversity indices expected heterozygosity (H_E_) and inbreeding coefficients (F_IS_) were calculated using the R packages “adegenet” and “hierfstat” (Goudet, 2005). The polymorphic information content (PIC) was calculated using an in-house R package. The two- and three-dimensional principal component analysis (PCA) was conducted using the R packages “stats” and “rgl”. A Bayesian clustering approach implemented in the program STRUCTURE version 2.3.4 (Pritchard et al., 2000) was used to analyse population genetic structure by setting replication number to 10,000 for the burn-in and Markov Chain Monte Carlo (MCMC) iterations each and using options of admixture model and correlated allele frequencies. The number of subpopulations, i.e., K was set from 1 to 7 and three independent runs were performed for each K. The Structure Harvester (https://taylor0.biology.ucla.edu/structureHarvester/) was used to analyze the results from the STRUCTURE software, which constructs a deltaK vs K plot using the method of Evanno et al. (2005). The weighted neighbour joining (NJ) was constructed in DARwin 6.0. (Perrier and Jacquemoud-Collet, 2006).

### Target site mutations in ACCase

Two primer pairs were used to amplify the ACCase gene sequence in *D. insularis*: FE35332 Forward (5′-ATG​TCC​ACT​CCT​GAA​TTC​CCA-3′), FE35333 Reverse (5′-CAT​TCT​GAG​GGA​AGT​ATC​AT-3′). PCR was performed in 25 μL reaction volume containing 5.0 µL of GoTaq Buffer, 0.5 µL of 10 mM dNTPs, 1.5 µL of 25 mM MgCl_2_, 0.5 µL of 10 µM of each forward and reverse primers, 0.2 µL of GoTaq G2 Hot Start Polymerase (Promega), and 14.8 µL of ultrapure nuclease-free water (Sigma). PCR cycling conditions were: one cycle at 95°C for 2 min, 35 cycles at 94°C for 30 s, 58°C for 30 s, 72°C for 90 s, and final extension at 72°C for 10 min. PCR product was run on 1.0% agarose gel to verify the amplicon size of 1.5 kb. The amplified samples were purified and sequenced in a Genetic Analyzer 3,500 instrument (Applied Biosystems, Thermo Fisher) following manufacturer’s instructions. Four individuals per population were sequenced using the original amplification primers and the following three internal sequencing forward primers: FE35334 Sequencing Forward 1 (5′- TGG​GAG​AGC​AAA​GCT​TGG​GGT-3′), FE35407 Sequencing Forward 2 (5′- GAA​GTG​CTG​CTA​TTG​CCA​GTG​C -3′) and FE35408 Sequencing Forward 3 (5′- GAC​CCA​CCA​GAC​AGA​CCT​GTT​A -3′). The chromatograms were manually read using Bioedit version 7.2.5 software (Hall, 1999) to screen the seven known target-site mutations.

### Genome wide association study (GWAS) and *in silico* analysis of significant SNPs

The normality of the resistance data scores was checked in PAST3 program (Hammer et al., 2001). The resistance scores data of haloxyfop at dose rate of 7.8 g a. i ha^−1^ and clethodim at dose rate of 27.0 g a. i ha^−1^ showed normal and near-normal distribution, respectively and hence were used in GWAS. Both general linear model (GLM) and mixed linear model (MLM) were used for GWAS in TASSEL software ver 5.0 (Bradbury et al., 2007). The kinship matrix was calculated using VanRaden algorithm (VanRaden, 2008) in the GAPIT package 2.0 (Lipka et al., 2012). In the GLM, PCA was used as a fixed variate and in the MLM, PCA and kinship matrices were used as fixed and random variates, respectively. The threshold to declare significant marker-trait associations (MTA) was ≥10^−3^ (log10p) after applying a correction for a false discovery rate (FDR) at *p* < 0.05.

The VCF file was annotated with SnpEff version 5.1 using the IWGC *D. insularis* reference genome and annotation v01.0. In addition, the *in silico* analysis of the significant SNPs was conducted using nucleotide Basic Local Alignment Search Tool (BLAST) in the EnsemblPlants database (https://plants.ensembl.org/index.html). The EnsemblPlants database has cDNA/transcript sequences of more than 80 monocots and dicots and of model plants, which were used to find the homologies. The genes found in the overlapping region and within 1.0 Mb upstream and downstream of the matched regions were selected as candidate genes. To determine their molecular functions, the protein sequences of the candidate genes were downloaded from EnsemblPlants database and used in protein BLAST analysis in NCBI server (https://blast.ncbi.nlm.nih.gov/Blast.cgi) and their molecular functions were determined after ascertaining their homologies with known proteins in grasses.

### Genomic prediction models

Four parametric models (Ridge regression, Bayes A, Bayes B, Bayes C) and a non-parametric model (RKHS) were used and all these models are implemented in the “BGLR” package (De Los Campos et al., 2022) in R. Ridge regression (RR) method considers common variance for all markers and shrinks the marker effects toward zero (Meuwissen et al., 2001). All Bayesian models do not consider the common variance of markers and incorporate additive genetic effects. The RKHS model uses a kernel function and captures non-additive effects (epistatic interactions). Either all available SNP markers were used or different sets of SNP markers were employed according to preliminary GWAS results. The SNP markers were ranked according to increasing *p*-values in GWAS analyses. Prediction accuracy of all models was calculated through “Pearson correlation coefficient” between observed and predicted values based on 100 iterations and 10-fold cross-validations.

## Results

### GBS marker distribution

**FIGURE 1:**
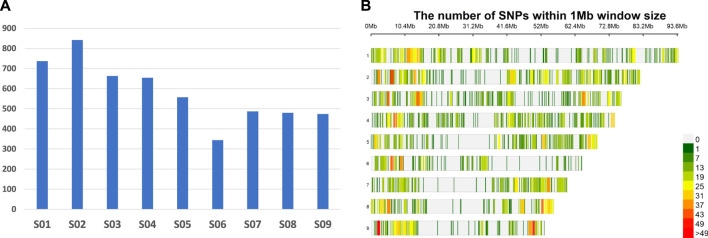
Genome wide GBS marker distribution of 5,238 SNPs; number of SNPs per chromosome **(A)** and SNP density per chromosome **(B)** (physical positions based on the current *Digitaria insularis* reference genome in Weedpedia).

A total of 105,699 single nucleotide polymorphisms (SNPs) were generated across 205 populations using the criterion of a minimum coverage to call a SNP. Of these, 5,238 SNPs were retained for genetic analysis after filtering for 5% minor allele frequency (MAF) and missing data 30% and removing markers that could not be mapped on the currently available reference genome of *D. insularis* on Weedpedia (https://weedpedia.weedgenomics.org/). The polymorphic information content (PIC) of the filtered SNPs varied from 0.10 to 0.38. Overall, SNPs showed good distribution with chromosomes S02 and S06 showing the highest (842) and the least (344) number of SNPs, respectively (Figure 1). On an average, 624 Mb of the physical genome was encompassed by these SNPs with the maximum genome coverage achieved on chromosome S01 (93.7 Mb) and minimum (51.9 Mb) on chromosome S09 (Supplementary Table S2).

### Within populations genetic diversity and population differentiation

The expected heterozygosity (H_E_) and polymorphic information content (PIC) values of the populations ranged from 0.56 to 0.64 and 0.20 to 0.37, with an average of 0.62 and 0.37, respectively (Table 1). Both parameters, H_E_ and PIC, showed that Minas Gerais (MG) population was moderately diverse while most other populations showed high level of genetic diversity (H_E_ > 0.60). The inbreeding coefficient (F_IS_) for all the seven populations was negative (Table 1) suggesting highly outcrossing nature of the populations under local environmental conditions.

**TABLE 1:** Expected heterozygosity (H_E_), polymorphic information content (PIC) and inbreeding coefficient (Fis) of *Digitaria insularis* populations from Mato Grosso (MT), Mato Grosso do Sul (MS), Minas Gerais (MG), Bahia (BA), Goiás (GO), Paraná (PR) and Rio Grande do Sul (RS).

Population	Number of populations sampled	H_E_	PIC	Fis
MT	74	0.63	0.36	−0.58
MS	10	0.64	0.35	−0.57
MG	24	0.56	0.20	−0.78
BA	24	0.62	0.32	−0.62
GO	21	0.63	0.35	−0.59
PR	35	0.63	0.37	−0.58
RS	18	0.63	0.36	−0.58
Overall	201	0.62	0.37	−0.61

The average Fst across all samples s was 0.020, indicating a low genetic differentiation among populations. The pairwise Fst values ranged from 0.003 (between populations MS and PR, GO and PR, MT and RS and PR and RS) to 0.064 (between MG and BA) (Table 2). As expected, a high gene flow (Nm) was detected among populations, varying from 3.65 to 83.08, with an overall average of 12.25. Interestingly, the lowest Nm estimates were obtained between MG and five populations (MT, MS, PR, RS and BA) (Table 2).

**TABLE 2:** Genetic differentiation coefficient (Fst) and gene flow (Nm) between populations. The upper and lower triangles represent pairwise Fst and Nm values, respectively.

	MT	MS	MG	BA	GO	PR	RS
MT		0.005	0.040	0.004	0.007	0.008	0.003
MS	49.75		0.042	0.004	0.004	0.003	0.005
MG	6.00	5.70		0.064	0.009	0.024	0.036
BA	62.25	62.25	3.65		0.019	0.006	0.008
GO	35.46	62.25	27.52	12.90		0.003	0.004
PR	31.00	83.08	10.16	41.41	83.08		0.003
RS	83.08	49.75	6.70	31.00	62.25	83.08	

### Principal component analysis (PCA) and STRUCTURE analysis

**FIGURE 2:**
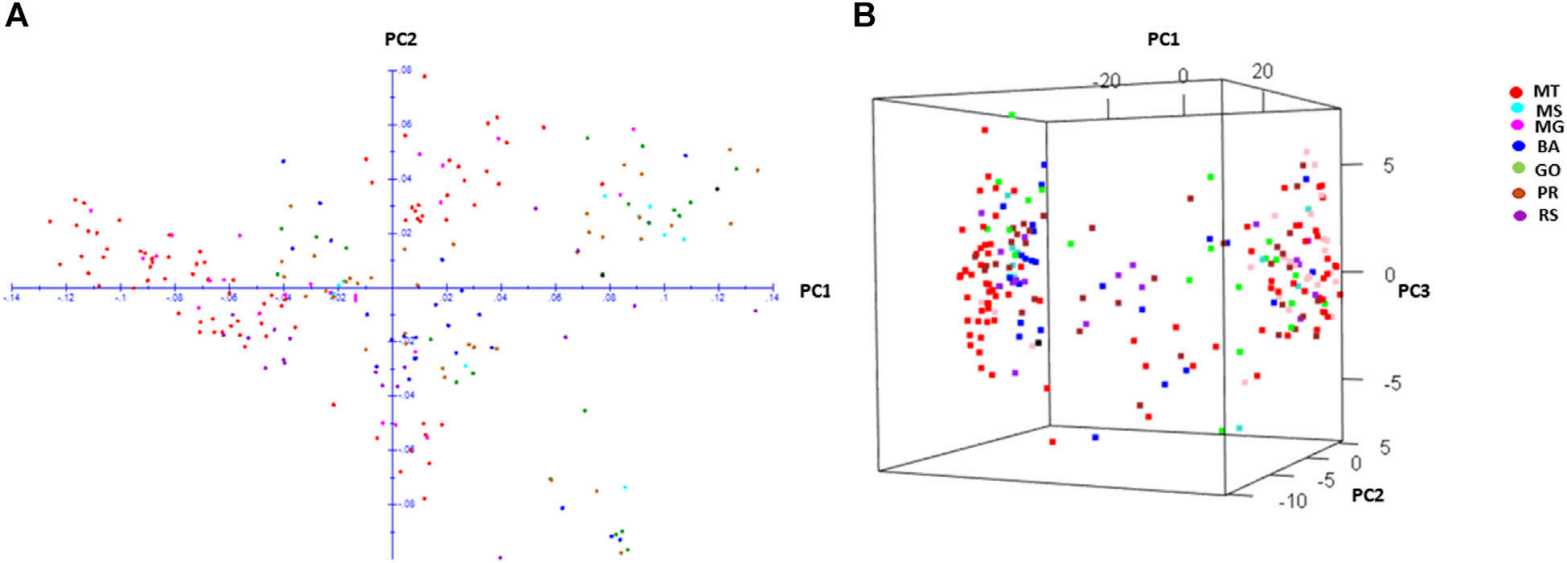
Two-dimensional **(A)** and three-dimensional **(B)** principal component analysis of *Digitaria insularis* populations based on 5,238 SNPs.

The PCA plot with genome wide 5,238 SNPs revealed two important features; i) all populations were scattered in the PCA plot and ii) populations did not show clusters based on their geographical origin (Figure 2). The population structure explored by Bayesian STRUCTURE analysis revealed two subpopulations at the best K (K = 2) (Supplementary Figures S1A, B). Both subpopulations comprised of individuals from all seven populations (Supplementary Figure S1B). Only populations from MG had a defined cluster (cluster II) based on a cluster membership threshold of 0.80 (Supplementary Figure S1B), whereas all remaining populations were distributed between the two clusters. The weighted neighbour joining (NJ) tree confirmed that two groups comprised individuals from seven populations clustered randomly irrespective of their geographic origin (Supplementary Figure S2).

**FIGURE 3:**
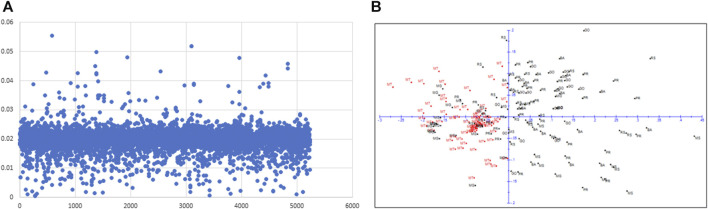
Plot of Fst outlier analysis showing 63 SNPs Fst>0.03 **(A)** and 2D-PCA with 63 SNPs showing a moderate differentiation of MT population from rest of the populations **(B)**.

With the Fst outlier analysis, we detected 63 SNPs that showed Fst values higher than the average Fst (Figure 3A). We used these 63 SNPs to redraw PCA to investigate if any geographical differentiation could be observed (Figure 3B). Interestingly, using a subset of 63 SNPs (Fst >0.03), MT population could be moderately differentiated from rest of the populations while the remaining six populations remained mixed (Figure 3B). To understand the functional significance of this subtle geographical differentiation, we conducted an *in silico* analysis of the sequences of the top fifteen outlier hits (Fst >0.04) to identify candidate genes (Supplementary Table S3). It was revealed that orthologs of the genes involved in growth and development and/or stress pathways showed hits with these sequences.

### Resistance to ACCase inhibitors and population structure of resistant and sensitive populations

As part of the resistance monitoring program, the *D. insularis* populations were tested with, haloxyfop at 7.8, 15.0, 27.0 and 64.0 g a. i ha^−1^ ai/ha and clethodim at 27, 54 and 108 g a. i ha^−1^ ai/ha. A total of 29 (15.0%), 29 (15.0%), 7 (3.6%) and 6 (3.1%) populations were found to show less than 90% control with haloxyfop at 7.8, 15.0, 27.0 and 64.0 g a. i ha^−1^, respectively. For clethodim, 16 (8.2%), 8 (4.1%) and 4 (2.0%) populations were found to be controlled at less than 90% at doses 27.0, 54.0 and 108.0 g a. i ha^-1^, respectively. There were no significant differences in the sensitivity scores for the two lower doses of haloxyfop, i.e., 7.8 and 15.0 g a. i ha^−1^, hence sensitivity scores from the dose 7.8 g a. i ha^−1^ were used in all further analysis. The state wise distribution showed that more than 50% of populations with reduced sensitivity to the haloxyfop dose rate 7.8 g a. i ha^-1^ were from MG state followed by MT (31%) (Supplementary Figure S3A). For higher doses of haloxyfop and for all the three doses of clethodim, populations with reduced sensitivity were from PR and MS states (Supplementary Figure S3B). The efficacy scores at 7.8 g a. i ha^−1^ dose of haloxyfop and 27.0 g a. i ha^−1^ of clethodim showed a normal and near-normal distribution, respectively (Supplementary Figures S3C, D).

Sequencing of the ACCase gene in all samples revealed that only two populations (BR-20-Din-144 and BR-21-Din-046) showed the presence of Trp2027Cys mutation. Whilst both populations showed reduced sensitivity to all the four doses of haloxyfop (7.8, 15.0, 27.0 and 64.0 g a. i ha^−1^), only one exhibited reduced sensitivity to the two doses (27.0 and 54.0 g a. i ha^−1^) of clethodim. The four populations (BR-21-Din-417, BR-21-Din-419, BR-21-Din-421 and BR-21-Din-423), that showed reduced sensitivity to the higher doses of haloxyfop (27.0 and 64.0 g a. i ha^−1^) and to all the three doses of clethodim (27.0, 54.0 and 108.0 g a. i ha^−1^), did not show the Trp2027Cys mutation. None of the other known mutations in this gene were identified in the resistant populations.

**FIGURE 4:**
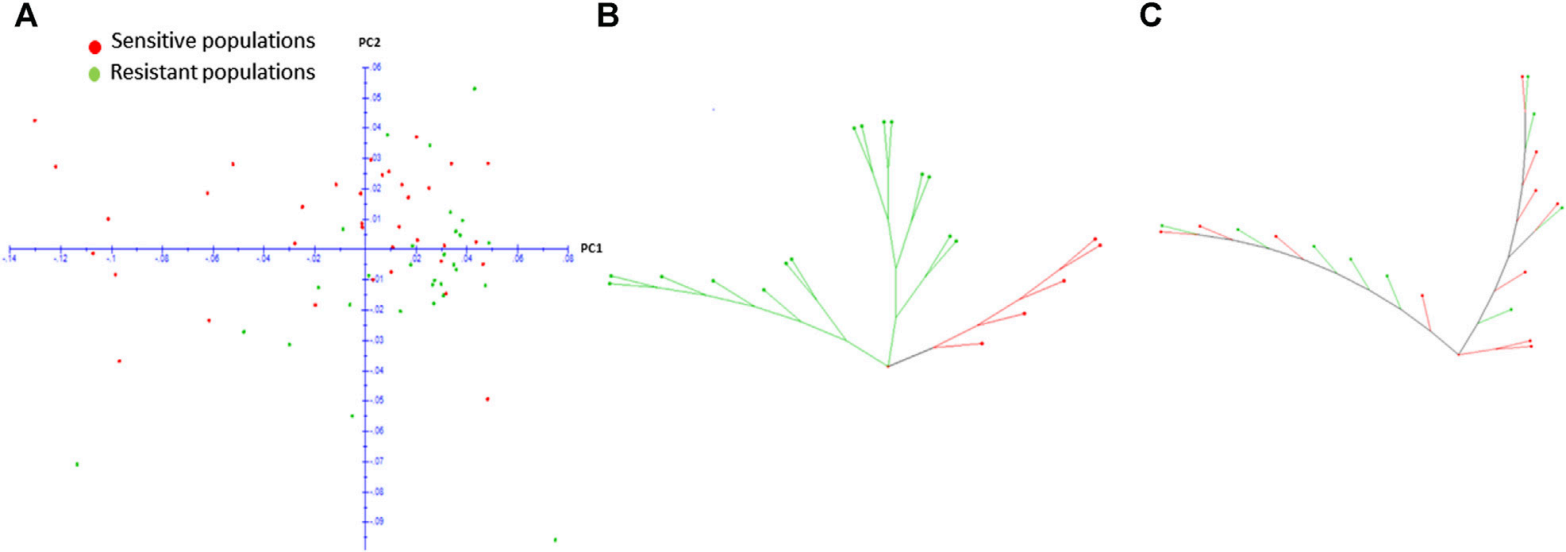
Two-dimensional PCA plot of resistant and sensitive populations from different geographic regions in Brazil **(A)** and weighted NJ trees of resistant and sensitive populations from Minas Gerais **(B)** and Mato Grosso **(C)** based on complete set of 5,238 SNPs.

To understand whether the high gene flow observed in the species is shaping the genetic structure of resistance, we investigated the population genetic structure of a subset of 65 populations, contrasting for resistance or reduced sensitivity to 7.8 g a. i ha^−1^ of haloxyfop regardless of geographic origin. Only 7.8 g a. i ha^−1^ dose rate was selected for these analyses as this dose rate showed a good size of populations with reduced sensitivity (29) which could be compared with sensitive populations (36). The other dose rates produced too few populations showing reduced sensitivity (<8) and hence were not analysed further. The PCA plot showed that resistant and sensitive populations were interspersed, and no clear groups were observed (Figure 4A), which led us to suggest that the resistance has evolved independently across the landscape. We then drew weighted NJ trees of populations from MG and MT regions separately (Figures 4B, C). For MG, the tree showed distinction between resistant and sensitive populations but for MT, resistant and sensitive populations were interspersed.

### Genome wide association study (GWAS) and genomic prediction models

**FIGURE 5:**
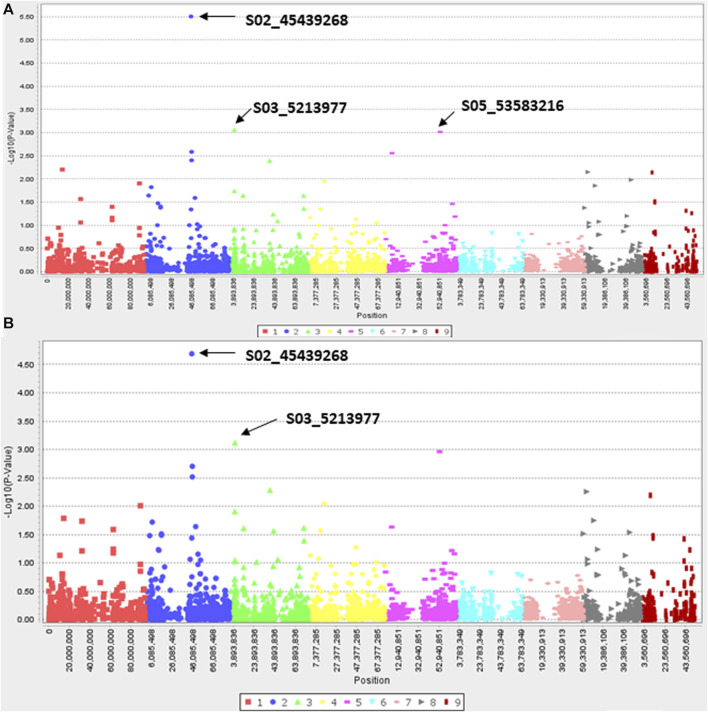
Manhattan plots showing marker-trait associations identified for resistance to Haloxyfop in Digitaria insularis with GLM **(A)** and MLM **(B)** models.

Since efficacy scores at 7.8 g a. i ha^−1^ dose of haloxyfop and 27.0 g a. i ha^−1^ of clethodim showed a normal and near-normal distribution, respectively (Supplementary Figures S3C, D), characteristics of a quantitative trait, a pilot GWAS study was conducted to identify candidate genes associated with reduced efficacy to haloxyfop and clethodim. Three and two SNPs were found associated with reduced efficacy with haloxyfop in GLM and MLM models, respectively (Figure 5). Two SNPs, S02_45439268 and S03_5213977, were common in both models (Table 3). S02_45439268 on chromosome 2 explained the highest percentage variation (*R* ^2^) of 26.6% (*p*-value = 3.13E-06), while S03_5213977 on chromosome 3 explained 14.6% *R* ^2^ (*p*-value = 9.71E-04). *In silico* analysis (BLAST analysis using reference genomes/cDNA sequences of *D. exilis* or model species) of these SNPs revealed that the SNP S02_45439268 was located 0.1 Mb upstream of a *D. exilis* gene *Dexi2A01G0009650*. The protein BLAST analysis of the protein sequence of this gene showed homologies with UDP-glycosyltransferase 91C1-like protein (UDPGT91C1) of *Panicum virgatum*, *Sorghum bicolor*, *Oryza glaberrima* and *Setaria italica*. The SNP S03_5213977 showed homologies with the galactinol--sucrose galactosyltransferase (GT) 2 in *Hordeum vulgare*, *Aegilops tauschii*, *Triticum urartu* and *Lolium arundinaceum*.

**TABLE 3:** Marker-trait associations identified by genome wide association study for resistance to Haloxyfop and clethodim and candidate gene hits.

Trait	Marker	Chr	Position	*p*-Value	*R* ^2^ (x 100)	Candidate gene hits
Resistance to haloxyfop	S02_45439268^a^	2	45439268	3.13E-06	0.26682	UDPGT91C1
	S03_5213977^a^	3	5213977	8.72E-04	0.14603	GT 2
	S05_53583216	5	53583216	9.71E-04	0.13438	
Resistance to clethodim	S09_10317698	9	10317698	2.35E-07	0.16425	
	S02_43460448	2	43460448	6.38E-06	0.1148	
	S05_47637569	5	47637569	7.57E-06	0.11327	VPS20
	S02_6916309	2	6916309	9.18E-06	0.12125	
	S08_15270772	8	15270772	2.36E-04	0.24071	

^a^ SNPs, identified in both GLM, and MLM.

**FIGURE 6:**
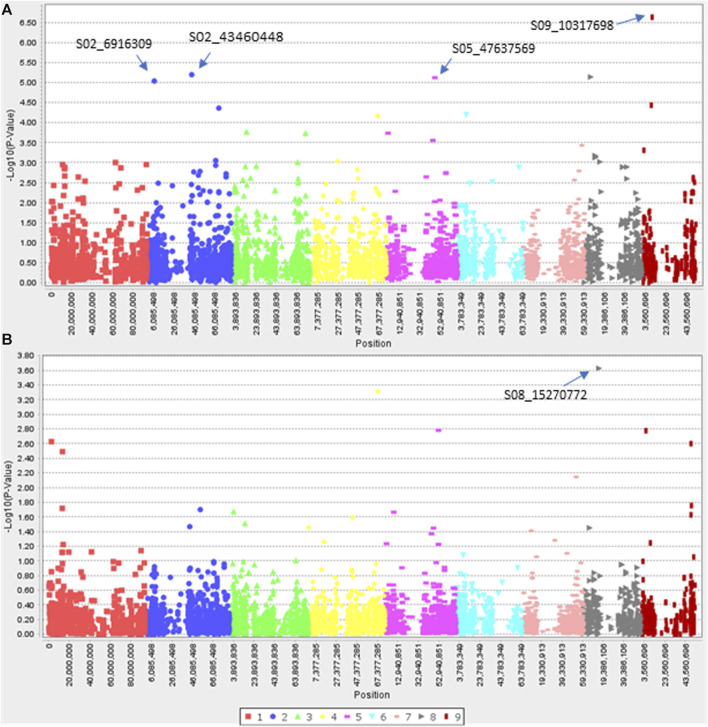
Manhattan plots showing marker-trait associations identified for resistance to Clethodim.

For efficacy shift to clethodim, four and one SNP were identified with GLM and MLM, respectively (Figure 6). The four SNPs with the GLM model explained 11.3%–16.6% of *R* ^2^, while the SNP identified with the MLM explained 24.0% of *R* ^2^ (Table 3). Interestingly, two SNPs were located within 0.1 Mb of potential NTSR genes. The SNP S05_47637569 was located within 0.1 Mb of *D. exilis* gene *Dexi1A01G0019350*. The BLASTP analysis of protein sequence of this gene showed homologies with vacuolar protein sorting (VPS)-associated protein 20 of *P. virgatum*, *S. italica*, *Zea mays* and *Lolium rigidum*. The SNP S08_15270772 is located within 0.1 Mb of *D. exilis* gene *Dexi9A01G0037640*, the protein sequence of which showed homologies with CMP-sialic acid transporter 2 of *S. italica*, *P. virgatum* and *S. bicolor*.

**FIGURE 7:**
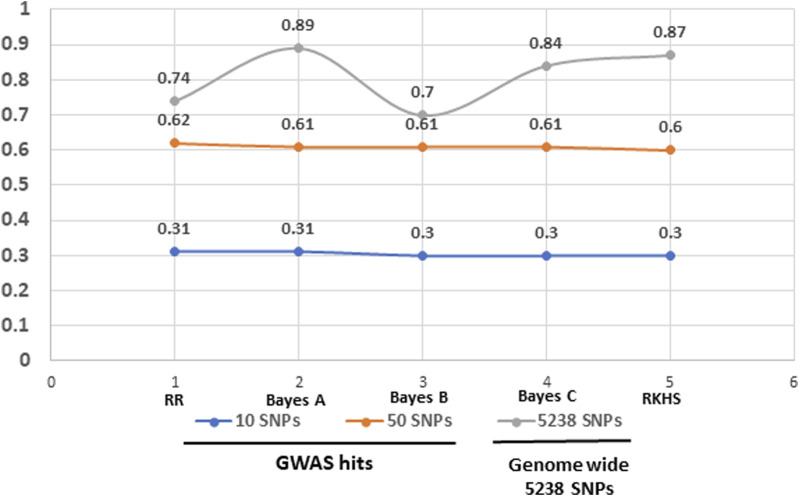
Prediction accuracy of five genomic prediction models tested using a subset of GWAS.

We further investigated the potential of genomic prediction (GP) models to predict NTSR resistance in the panel using resistance data scores at haloxyfop dose of 7.8 g a. i ha^-1^. We tested five models, Ridge Regression, Bayes A, Bayes B, Bayes C and RKHS, on different subsets of SNPs selected either based on GWAS hits or complete set of 5,238 SNPs (Figure 7). Although predictions based on models having 50 SNPs from GWAS showed more than 50% prediction accuracy, the highest accuracy was achieved with complete set of 5,238 SNPs with better prediction accuracy from Bayes A or RKHS models.

## Discussion

The present study highlights the outcomes of the assessment of neutral genetic variation in *D. insularis* in its natural expansion range in Brazil, where it has recently become one of the most problematic agricultural weeds. Both genetic diversity parameters (PIC and H_E_) indicated an overall high genetic diversity in the populations. The H_E_ estimates in populations (0.56–0.64) are higher than the previously reported values for this species by GBS markers (0.15–0.45; Netto et al., 2021). This discrepancy is partly due to differences in the sample size, i.e., a smaller set analysed in the previous study and due to the fact that only glyphosate resistant and susceptible populations were investigated earlier (Gonçalves Netto et al., 2021). A further comparison with other outcrossing weed species revealed higher genetic diversity in *D. insularis* as compared to *Alopecurus myosuroides* (average H_E_ = 0.24) and *Ipomoea purpurea* (average H_E_ = 0.24) and lower genetic diversity than *Lolium multiflorum* (average H_E_ = 0.81) (Délye et al., 2010; Kuester et al., 2015; Karn and Jasieniuk, 2017; Dixon et al., 2021).

The PCA, STRUCTURE and NJ tree analyses based on neutral GBS markers unveiled the absence of geographic differentiation among populations and concurrently reiterated the fact that geographical distance does not govern the genetic structure of *D. insularis* across the landscape (Figure 2). This lack of spatial patterning has been revealed in several weed species that are outcrossing and possess a high seed dispersal ability such as black grass and common morning glory (Kuester et al., 2015; Dixon et al., 2021). The same is true for invasive weed species that have shown recent expansion such as common ragweed (Martin et al., 2016) and *M. micrantha* (Ruan et al., 2021). Several factors are associated with the lack of structure or spatial patterning observed in weeds, including their outcrossing nature leading to higher gene flow between populations as compared to inbreeding species, human-mediated modes of dispersal (e.g., dispersal through farm machinery) and their recent expansion across the landscape (Kuester et al., 2015). The population genetic differentiation coefficient (Fst) confirmed a very low genetic differentiation between *D. insularis* populations (0.003 ≤ F_ST_ ≤ 0.064), suggesting that gene flow between populations was common. The interpopulation gene flow estimates (Nm = 3.65–83.08) were approximately two-fold higher than the values obtained in invasive weed *Mikania micrantha* (Ruan et al., 2021). Such low level of population differentiation and high level of gene flow among populations are indicative of recent expansion of the species as an agricultural weed in Brazil.

We also hypothesized that the prevailing agricultural practices in Brazil have played a significant role in shaping the genetic structure of *D. insularis* populations investigated here. As a common practice, many Brazilian soybean growers own many large-scale farms, serving urban and rural markets, across the country and transport of combines is common between the farms during harvesting (Lopez Ovejero et al., 2017). Through hair like appendages, *D. insularis* seeds adhere to combines, leading to an increased genetic exchange between different populations thereby weakening population divergence. This also explains the high gene flow detected between *D. insularis* populations collected from southern and mid-western states (Table 2).

The Fst outlier analysis detected 63 SNPs that showed Fst values above genome-wide average obtained with 5,238 SNPs. Using these SNPs in PCA evidenced a subtle genetic structure; populations from MT could be moderately differentiated from the remaining populations (Figure 3B). The *in silico* analysis of the top outlier loci revealed hits with adaptive genes such as DUF579, DUF2404 and GRAS family transcription factor involved in growth and development and/or abiotic stress responses (Lee et al., 2012; Wang et al., 2022). In invasive weed species, adaptive signatures throughout the genomes have been identified using GBS markers and SNPs active in geographic differentiation detected more often pathways related to growth and defence responses (Martin et al., 2016; van Boheemen et al., 2017; Ruan et al., 2021). The identification of similar functional genes in the present study therefore suggests that the populations have been adapting to changes in the environments while going through population expansion. There are evidences that indicate that the populations from southern, south-eastern, and north-eastern states (PR, RS, MS, MG, BA) have been exposed to cyclical droughts more often than populations from mid-western states such as MT (Nobre et al., 2016; Cunha et al., 2018; Marengo et al., 2020).

Two different models of resistance evolution have been proposed in weed species, i.e., gene flow and independent evolution (Kuester et al., 2015; Délye et al., 2010). Currently, only a limited number of studies have addressed the issue by simultaneous assessment of neutral genetic variation with the level of resistance in weed species across the spatial scale (Küpper et al., 2018; Dixon et al., 2021). Such studies are required in more weed species, both inbreeding and outcrossing, to provide insights into the evolutionary units of herbicide resistance. By combining neutral genetic variation with the level of resistance, if a pattern of isolation-by-distance (IBD) is displayed, we can infer that resistance has spread by gene flow in a weed species. In the second scenario, if a mosaic resistance pattern is exhibited and no evidence of IBD across populations, it would suggest independent evolution of resistance on a local scale or at larger distances. Such studies will prove of high relevance to applied weed scientists willing to maintain low levels of resistance as these will help to make informed decisions about weed management strategies, which would differ in the two scenarios.

We investigated, using a subset of populations showing susceptibility and reduced sensitivity to haloxyfop, whether the potential for spread of resistance occurs through gene flow or through independent evolution in this weed species. Although we did see a moderate differentiation between the resistant and sensitive populations, a mosaic pattern of resistance, i.e., resistant populations placed closely to sensitive populations, was generally observed across the landscape in the PCA (Figure 4A). The weighted NJ trees of the local resistant and sensitive populations within MT and MG states (Figures 4B, C) provided explicit evidence that resistance is evolving by both means (gene flow and independent evolution) in *D. insularis*. Within MG, two clusters of resistant populations were evident which were separated from the sensitive populations (Figure 4B), whereas within MT a mosaic pattern was evident (Figure 4C). These results suggests that both evolutionary mechanisms, gene flow and multiple, independent resistance evolution, are contributing to the evolution of resistance in *D. insularis*. This contrasts with the results obtained in black grass (Délye et al., 2010; Dixon et al., 2021) and common morning glory (Kuester et al., 2015) that reported only independent evolution of resistance across the landscape.

The sequencing of the ACCase gene to screen the seven known target-site mutations (Powles and Yu, 2010; Kaundun et al., 2021) showed the presence of Trp2027Cys mutation in two populations only. It is noteworthy that these two populations showed clear survivorship at field rate (64.0 g a. i ha^−1^) of haloxyfop, consistent with the impact of the W2027C mutation on FOP herbicides (Kaundun, 2014). A large majority of populations showing reduced sensitivity to lower doses of haloxyfop (7.8 or 15.0 g a. i ha^−1^) or clethodim (27.0 g a. i ha^−1^) did not show this or other known target-site mutations. In the light of the present results, although it seems safe to assume that NTS mechanisms are likely the cause of reduced sensitivity of 15% of populations, further studies including sequencing of the ACCase gene on hundreds of individuals of these populations will be required to rule out the possibility of co-existence of TSR (at a very low frequency) and NTSR. It is important to note that previous studies in weeds reporting TSR as the leading mechanism of resistance to ACCase inhibitors largely involved smaller number of populations and only field dose rates, which precluded the plausibility of detection of NTSR genes.

GWAS analysis for haloxyfop resistance identified two important SNPs that together explained 41.0% of variation. One of these SNPs (S03_5213977) showed hits with galactinol--sucrose galactosyltransferase (GT) 2 of multiple grass species including *H. vulgare*, *A. tauschii*, *T. urartu* and *L. arundinaceum*. GTs are a well-researched group of NTSR enzymes known for herbicide detoxification in several weed species such as *L. rigidum* (Gaines et al., 2014; Duhoux et al., 2015), *A. myosuroides* (Gardin et al., 2015) and *Eleusine indica* (Chen et al., 2015). The second SNP (S02_45439268) for reduced sensitivity to haloxyfop was located within 0.1 Mb upstream of a gene *Dexi2A01G0009650* in *D. exilis* (reference genome of a cultivated species of *Digitaria*) encoding a putative NTSR enzyme UDP-glycosyltransferase 91C1-like protein (UDPGT91C1), the role of which in herbicide detoxification mechanisms has been shown only recently in *Arabidopsis thaliana* through its glucosylating activity (Huang et al., 2021). Using both mutant and overexpression lines, it was demonstrated that UDPGT91C1 can glycosylate the herbicide sulcotrione, a triketone herbicide. UDPGTs (also known as UGTs), in addition to CYP450s and GSTs, have been implicated in herbicide detoxification by metabolism, and upregulated expression of some UDPGT genes has been observed with degradation of herbicides (Matzrafi et al., 2017; Vega et al., 2020; Huang et al., 2021; Chandra and Leon, 2022).

For reduced sensitivity to clethodim, two important candidate gene hits with multiple grass species were obtained in close vicinities of two significant SNPs (S05_47637569 and SNP S08_15270772). Of these, vacuolar protein sorting (VPS)-associated protein 20 (VPS20) is part of Endosomal Sorting Complex Required for Transport (ESCRT)-III, responsible for vacuolar-based degradation pathway (Agaoua et al., 2021). The herbicides that are inactivated by GST-catalyzed glutathione conjugation are transported into vacuoles for further metabolism (YU et al., 2013; Cai et al., 2014). A VPS60-type candidate target gene has been selected for validation in relation to NTS mechanism of glyphosate resistance mechanism in Lolium multiflorum (Cechin et al., 2020).

Finally, we tested five genomic models; Ridge Regression, Bayes A, Bayes B, Bayes C and RKHS, to explore the ability of these models for predicting NTSR in *D. insularis*. While Ridge Regression model assumes that all markers have same effects, Bayesian models (Bayes A, Bayes B and Bayes C) incorporate additive genetic effects and RKHS captures complex epistatic interactions. As anticipated, we got reasonably high prediction with the reduced set of SNPs selected by GWAS (the case of 50 GWAS SNPs), comparable to the whole set of 5,238 SNPs (Figure 7). All models invariably showed ≥0.60 prediction accuracy with the 50 SNPs selected by GWAS as compared to 10 SNPs. However, the highest prediction power (≥0.80) was achieved with the complete set of 5,238 SNPs with the models Bayes A and RKHS, incorporating SNPs with additive effects and epistatic interactions, respectively. These results contrast with the ones obtained by Dixon et al. (2021) in black grass, who obtained the highest prediction with the top few hundreds GWAS loci for resistance to fenoxaprop compared to using all markers.

## Conclusion

*Digitaria insularis* populations showed high genetic diversity and a population structure typical of a weed species that has shown recent expansion across the landscape. In most weed species, where resistance evolution to ACCase inhibitors has been investigated, it is indicated that resistance has evolved independently in weed populations. *Digitaria insularis*, on the other hand, is a fascinating example of herbicide resistance evolution, in which resistance has evolved by multiple mechanisms, i.e., gene flow and independent evolution. Since resistance to ACCase inhibitor haloxyfop is observed in Brazil, the identification of candidate genes conferring shifts in the sensitivity scores or reduced sensitivity to haloxyfop opens new opportunities to further investigate resistance mechanisms in this species and come up with effective weed management strategies. The present study also opens new avenues of functional validation of the candidate genes identified here to determine their role in NTSR-linked processes.

## Data Availability

The original contributions presented in the study are publicly available. The data have been submitted to the DRYAD database, https://doi.org/10.5061/dryad.9s4mw6mpt.

## References

[B1] Abou-Khater L. Maalouf F. Jighly A. Alsamman A. M. Rubiales D. Rispail N. (2022). Genomic regions associated with herbicide tolerance in a worldwide faba bean (Vicia faba L.) collection. Sci. Rep. 12, 158. 10.1038/s41598-021-03861-0 34996977 PMC8741826

[B2] Agaoua A. Bendahmane A. Moquet F. Dogimont C. (2021). Membrane trafficking proteins: a new target to identify resistance to viruses in plants. Plants 10, 2139. 10.3390/plants10102139 34685948 PMC8541145

[B3] Beckie H. J. Tardif F. J. (2012). Herbicide cross resistance in weeds. Crop Prot. 35, 15–28. 10.1016/j.cropro.2011.12.018

[B4] Bradbury P. J. Zhang Z. Kroon D. E. Casstevens T. M. Ramdoss Y. Buckler E. S. (2007). TASSEL: software for association mapping of complex traits in diverse samples. Bioinformatics 23, 2633–2635. 10.1093/bioinformatics/btm308 17586829

[B5] Cai Y. Zhuang X. Gao C. Wang X. Jiang L. (2014). The arabidopsis endosomal sorting complex required for transport III regulates internal vesicle formation of the prevacuolar compartment and is required for plant development. Plant Physiol. 165, 1328–1343. 10.1104/pp.114.238378 24812106 PMC4081340

[B6] Cechin J. Piasecki C. Benemann D. P. Kremer F. S. Galli V. Maia L. C. (2020). Transcriptome analysis identifies candidate target genes involved in glyphosate-resistance mechanism in lolium multiflorum. Plants 9, 685. 10.3390/plants9060685 32481698 PMC7357135

[B7] Chandra S. Leon R. G. (2022). Genome-wide evolutionary analysis of putative non-specific herbicide resistance genes and compilation of core promoters between monocots and dicots. Genes. (Basel) 13, 1171. 10.3390/genes13071171 35885954 PMC9316059

[B8] Chen S. McElroy J. S. Dane F. Peatman E. (2015). Optimizing transcriptome assemblies for eleusine indica leaf and seedling by combining multiple assemblies from three *de novo* assemblers. Plant Genome 8, 10.0064. 10.3835/plantgenome2014.10.0064 33228277

[B9] Cunha A. P. M. A. Tomasella J. Ribeiro-Neto G. G. Brown M. Garcia S. R. Brito S. B. (2018). Changes in the spatial–temporal patterns of droughts in the Brazilian Northeast. Atmos. Sci. Lett. 19. 10.1002/asl.855

[B10] de Carvalho L. B. Cruz-Hipolito H. González-Torralva F. da Costa Aguiar Alves P. L. Christoffoleti P. J. De Prado R. (2011). Detection of sourgrass (*Digitaria insularis*) biotypes resistant to glyphosate in Brazil. Weed Sci. 59, 171–176. 10.1614/WS-D-10-00113.1

[B11] De Los Campos G. Perez P. Maintainer R. Perez Rodriguez P. (2022). Title bayesian generalized linear regression.

[B12] Délye C. Michel S. Bérard A. Chauvel B. Brunel D. Guillemin J. (2010). Geographical variation in resistance to acetyl-coenzyme A carboxylase‐inhibiting herbicides across the range of the arable weed Alopecurus myosuroides (black-grass). New Phytol. 186, 1005–1017. 10.1111/j.1469-8137.2010.03233.x 20345631

[B13] Dixon A. Comont D. Slavov G. T. Neve P. (2021). Population genomics of selectively neutral genetic structure and herbicide resistance in UK populations of Alopecurus myosuroides. Pest Manag. Sci. 77, 1520–1529. 10.1002/ps.6174 33155426

[B14] Duhoux A. Carrère S. Gouzy J. Bonin L. Délye C. (2015). RNA-Seq analysis of rye-grass transcriptomic response to an herbicide inhibiting acetolactate-synthase identifies transcripts linked to non-target-site-based resistance. Plant Mol. Biol. 87, 473–487. 10.1007/s11103-015-0292-3 25636204

[B15] Evanno G. Regnaut S. Goudet J. (2005). Detecting the number of clusters of individuals using the software STRUCTURE: a simulation study. Mol. Ecol. 14, 2611–2620. 10.1111/j.1365-294X.2005.02553.x 15969739

[B16] Gaines T. A. Lorentz L. Figge A. Herrmann J. Maiwald F. Ott M. C. (2014). RNA-Seq transcriptome analysis to identify genes involved in metabolism-based diclofop resistance in Lolium rigidum. Plant J. 78, 865–876. 10.1111/tpj.12514 24654891

[B17] Gardin J. A. C. Gouzy J. Carrère S. Délye C. (2015). ALOMYbase, a resource to investigate non-target-site-based resistance to herbicides inhibiting acetolactate-synthase (ALS) in the major grass weed Alopecurus myosuroides (black-grass). BMC Genomics 16, 590. 10.1186/s12864-015-1804-x 26265378 PMC4534104

[B18] Gazziero D. L. P. Adegas F. S. Silva A. F. Concenco G. (2019). Estimating yield losses in soybean due to sourgrass interference. Planta Daninha 37. 10.1590/s0100-83582019370100047

[B19] Gonçalves Netto A. Cordeiro E. M. G. Nicolai M. de Carvalho S. J. P. Ovejero R. F. L. Brunharo C. A. C. G. (2021). Population genomics of Digitaria insularis from soybean areas in Brazil. Pest Manag. Sci. 77, 5375–5381. 10.1002/ps.6577 34302709 PMC9291757

[B20] Goudet J. (2005). HIERFSTAT, a package for R to compute and test hierarchical F-statistics. Mol. Ecol. Notes 5, 184–186. 10.1111/j.1471-8286.2004.00828.x

[B21] Hall T. A. (1999). BioEdit: a user-friendly biological sequence alignment editor and analysis program for Windows 95/98/NT. Nucleic Acids Symp. Ser. 41, 95–98.

[B22] Hammer Ø. Harper D. A. T. Ryan P. D. (2001). Past: paleontological statistics software package for education and data analysis. Palaeontol. Electron. 4, 351.

[B23] Han H. Yu Q. Owen M. J. Cawthray G. R. Powles S. B. (2016). Widespread occurrence of both metabolic and target-site herbicide resistance mechanisms in Lolium rigidum populations. Pest Manag. Sci. 72, 255–263. 10.1002/ps.3995 25703739

[B24] Heap I. (1999). “International survey of herbicide-resistant weeds: lessons and limitations,” in 1999 Brighton crop protection conference: weeds. Available at: https://www.cabdirect.org/cabdirect/abstract/20002301280 (Accessed November 14, 2023).

[B25] Huang X. xu Zhao S. man Zhang Y. ying Li Y. jie Shen H. nuo Li X. (2021). A novel UDP-glycosyltransferase 91C1 confers specific herbicide resistance through detoxification reaction in Arabidopsis. Plant Physiol. biochem. 159, 226–233. 10.1016/j.plaphy.2020.12.026 33387851

[B26] Iwakami S. Kamidate Y. Yamaguchi T. Ishizaka M. Endo M. Suda H. (2019). CYP 81A P450s are involved in concomitant cross‐resistance to acetolactate synthase and acetyl‐CoA carboxylase herbicides in *Echinochloa phyllopogon* . New Phytol. 221, 2112–2122. 10.1111/nph.15552 30347444

[B27] Karn E. Jasieniuk M. (2017). Genetic diversity and structure of Lolium perenne ssp. multiflorum in California vineyards and orchards indicate potential for spread of herbicide resistance via gene flow. Evol. Appl. 10, 616–629. 10.1111/eva.12478 28616068 PMC5469165

[B28] Kaundun S. S. (2014). Resistance to acetyl-CoA carboxylase-inhibiting herbicides. Pest Manag. Sci. 70, 1405–1417. 10.1002/ps.3790 24700409

[B29] Kaundun S. S. Downes J. Jackson L. V. Hutchings S. J. McIndoe E. (2021). Impact of a novel W2027L mutation and non-target site resistance on acetyl-CoA carboxylase-inhibiting herbicides in a French Lolium multiflorum population. Genes. (Basel) 12, 1838. 10.3390/genes12111838 PMC8620607 34828444

[B30] Kim C. Guo H. Kong W. Chandnani R. Shuang L.-S. Paterson A. H. (2016). Application of genotyping by sequencing technology to a variety of crop breeding programs. Plant Sci. 242, 14–22. 10.1016/j.plantsci.2015.04.016 26566821

[B31] Kuester A. Chang S. M. Baucom R. S. (2015). The geographic mosaic of herbicide resistance evolution in the common morning glory, Ipomoea purpurea: evidence for resistance hotspots and low genetic differentiation across the landscape. Evol. Appl. 8, 821–833. 10.1111/eva.12290 26366199 PMC4561571

[B32] Küpper A. Manmathan H. K. Giacomini D. Patterson E. L. McCloskey W. B. Gaines T. A. (2018). Population genetic structure in glyphosate-resistant and -susceptible palmer amaranth (Amaranthus palmeri) populations using genotyping-by-sequencing (GBS). Front. Plant Sci. 9, 29. 10.3389/fpls.2018.00029 29422910 PMC5788914

[B33] Lee C. Teng Q. Zhong R. Yuan Y. Haghighat M. Ye Z. H. (2012). Three arabidopsis DUF579 domain-containing GXM proteins are methyltransferases catalyzing 4-o-methylation of glucuronic acid on xylan. Plant Cell. Physiol. 53, 1934–1949. 10.1093/pcp/pcs138 23045523

[B34] Lipka A. E. Tian F. Wang Q. Peiffer J. Li M. Bradbury P. J. (2012). GAPIT: genome association and prediction integrated tool. Bioinformatics 28, 2397–2399. 10.1093/bioinformatics/bts444 22796960

[B35] Lopez Ovejero R. F. Takano H. K. Nicolai M. Ferreira A. Melo M. S. C. Cavenaghi A. L. (2017). Frequency and dispersal of glyphosate-resistant sourgrass (Digitaria insularis) populations across Brazilian agricultural production areas. Weed Sci. 65, 285–294. 10.1017/wsc.2016.31

[B36] Marengo J. A. Cunha A. P. M. A. Nobre C. A. Ribeiro Neto G. G. Magalhaes A. R. Torres R. R. (2020). Assessing drought in the drylands of northeast Brazil under regional warming exceeding 4 °C. Nat. Hazards 103, 2589–2611. 10.1007/s11069-020-04097-3

[B37] Martin M. D. Olsen M. T. Samaniego J. A. Zimmer E. A. Gilbert M. T. P. (2016). The population genomic basis of geographic differentiation in North American common ragweed (Ambrosia artemisiifolia L.). Ecol. Evol. 6, 3760–3771. 10.1002/ece3.2143 28725355 PMC5513308

[B38] Matzrafi M. Shaar-Moshe L. Rubin B. Peleg Z. (2017). Unraveling the transcriptional basis of temperature-dependent pinoxaden resistance in brachypodium hybridum. Front. Plant Sci. 8, 1064. 10.3389/fpls.2017.01064 28680434 PMC5478685

[B39] Meuwissen T. H. E. Hayes B. J. Goddard M. E. (2001). Prediction of total genetic value using genome-wide dense marker maps. Genetics 157, 1819–1829. 10.1093/genetics/157.4.1819 11290733 PMC1461589

[B40] Nobre C. A. Marengo J. A. Seluchi M. E. Cuartas L. A. Alves L. M. (2016). Some characteristics and impacts of the drought and water crisis in southeastern Brazil during 2014 and 2015. J. Water Resour. Prot. 08, 252–262. 10.4236/jwarp.2016.82022

[B41] Perrier X. Jacquemoud-Collet J. P. (2006). Darwin software. Available at: https://darwin.cirad.fr/darwin.

[B42] Poland J. A. Rife T. W. (2012). Genotyping‐by‐Sequencing for plant breeding and genetics. Plant Genome 5. 10.3835/plantgenome2012.05.0005

[B43] Powles S. B. Yu Q. (2010). Evolution in action: plants resistant to herbicides. Annu. Rev. Plant Biol. 61, 317–347. 10.1146/annurev-arplant-042809-112119 20192743

[B44] Pritchard J. K. Stephens M. Donnelly P. (2000). Inference of population structure using multilocus genotype data. Genetics 155, 945–959. 10.1093/genetics/155.2.945 10835412 PMC1461096

[B45] Ruan X. Wang Z. Su Y. Wang T. (2021). Population genomics reveals gene flow and adaptive signature in invasive weed mikania micrantha. Genes. (Basel) 12, 1279. 10.3390/genes12081279 34440453 PMC8394975

[B46] Sehgal D. Autrique E. Singh R. Ellis M. Singh S. Dreisigacker S. (2017). Identification of genomic regions for grain yield and yield stability and their epistatic interactions. Sci. Rep. 7, 41578. 10.1038/srep41578 28145508 PMC5286416

[B47] Sehgal D. Rosyara U. Mondal S. Singh R. Poland J. Dreisigacker S. (2020). Incorporating genome-wide association mapping results into genomic prediction models for grain yield and yield stability in CIMMYT spring bread wheat. Front. Plant Sci. 11, 197. 10.3389/fpls.2020.00197 32194596 PMC7064468

[B48] Shi C. Zheng Y. Geng J. Liu C. Pei H. Ren Y. (2020). Identification of herbicide resistance loci using a genome-wide association study and linkage mapping in Chinese common wheat. Crop J. 8, 666–675. 10.1016/j.cj.2020.02.004

[B49] van Boheemen L. A. Lombaert E. Nurkowski K. A. Gauffre B. Rieseberg L. H. Hodgins K. A. (2017). Multiple introductions, admixture and bridgehead invasion characterize the introduction history of Ambrosia artemisiifolia in Europe and Australia. Mol. Ecol. 26, 5421–5434. 10.1111/mec.14293 28802079

[B50] VanRaden P. M. (2008). Efficient methods to compute genomic predictions. J. Dairy Sci. 91, 4414–4423. 10.3168/jds.2007-0980 18946147

[B51] Vega T. Gil M. Martin G. Moschen S. Picardi L. Nestares G. (2020). Stress response and detoxification mechanisms involved in non-target-site herbicide resistance in sunflower. Crop Sci. 60, 1809–1822. 10.1002/csc2.20138

[B52] Wang S. Duan Z. Yan Q. Wu F. Zhou P. Zhang J. (2022). Genome−Wide identification of the GRAS family genes in melilotus albus and expression analysis under various tissues and abiotic stresses. Int. J. Mol. Sci. 23, 7403. 10.3390/ijms23137403 35806414 PMC9267034

[B53] Yu Q. Han H. Cawthraya G. R. Wang S. F. Powles S. B. (2013). Enhanced rates of herbicide metabolism in low herbicide‐dose selected resistant Lolium rigidum. Plant. Cell. Environ. 36, 818–827. 10.1111/pce.12017 23046181

