# Supplementary material for: Genomic characterisation and dissection of the onset of resistance to acetyl CoA carboxylase-inhibiting herbicides in a large collection of Digitaria insularis from Brazil
Source: Front Genet. 2024 Feb 19;15:1340852. doi: 10.3389/fgene.2024.1340852 (PMC10910277; doi:10.3389/fgene.2024.1340852)
Supplement: Supplementary file 1 [file DataSheet1.PDF]

Genomic characterisation and dissection of the onset of resistance to acetyl CoA carboxylase-inhibiting herbicides in a large collection of *Digitaria insularis* from Brazil

Deepmala Sehgal[1,*], Claudia Oliveira[2], Sandra Mathioni[2], Stephanie Widdison[1], Will Plumb[1], Breno Campos[1], Shiv Shankar Kaundun[1,*]

1. Syngenta Ltd., Jealott's Hill International Research Centre, Bracknell, Berkshire, RG42 6EY, UK
2. Syngenta Crop Protection, Holambra Research and Development Center, Estrada Municipal HBR-333, Holambra, São Paulo, Brazil, 13.825-000

Corresponding authors

deepmala.sehgal@syngenta.com

deepak.kaundun@syngenta.com

Table S1 *D. insularis* biotypes from Brazil used in the present study

S. no	Sample ID	Origin state	Year of collection	Crop field	Latitude	First herbicide treatment	Second herbicide treatment	Third herbicide treatment
1	BR-20-Din-001	Mato Grosso	2020	Soybean	-16.31607	Fluroxypyr	Glyphosate	Chlorimuron
2	BR-20-Din-002	Mato Grosso	2020	Soybean	-16.270551	Glyphosate	Chlorimuron	Clethodim
3	BR-20-Din-144	Mato Grosso	2020	Soybean	-15.57713	Glyphosate	Flumioxazina	Glyphosate
4	BR-21-Din-026	Mato Grosso	2021	Soybean	-14.620445	Glyphosate	2,4-D	Glyphosate
5	BR-21-Din-119	Mato Grosso	2021	Soybean	-13.011434	Clethodim	Haloxyfop	Imazethapyr
7	BR-21-Din-172	Mato Grosso	2021	Soybean	-15.384962			
8	BR-21-Din-178	Mato Grosso	2021	Soybean	-14.596689	Glyphosate	Clethodim	Glyphosate
9	BR-21-Din-188	Mato Grosso	2021	Soybean	-14.344837			
10	BR-21-Din-029	Mato Grosso	2021	Soybean	-13614090	Glyphosate	2,4-D	Glyphosate
11	BR-21-Din-072	Mato Grosso	2021	Soybean	-13.26769			
12	BR-21-Din-134	Mato Grosso	2021	Soybean	-12.339292			
13	BR-21-Din-140	Mato Grosso	2021	Soybean	-12.514377			
14	BR-21-Din-136	Mato Grosso	2021	Soybean	-12.823289			
15	BR-21-Din-133	Mato Grosso	2021	Soybean	-12.104671	Glyphosate	Imazethapyr	Chlorimuron
16	BR-21-Din-086	Mato Grosso	2021	Soybean	-13.867996	Glyphosate	Clethodim	
17	BR-21-Din-076	Mato Grosso	2021	Soybean	-12.63244	Glyphosate	2,4-D	Glyphosate
18	BR-21-Din-080	Mato Grosso	2021	Soybean	-12.879616	S-metolachlor	Glyphosate	Glyphosate
19	BR-21-Din-081	Mato Grosso	2021	Soybean	-13.803954	S-metolachlor	Glyphosate	Clethodim
20	BR-21-Din-009	Mato Grosso	2021	Soybean	-16.857539			
21	BR-21-Din-012	Mato Grosso	2021	Soybean	-17.227875			
22	BR-21-Din-185	Mato Grosso	2021	Soybean	-14.600997	Glyphosate	Paraquat	Glyphosate
23	BR-21-Din-010	Mato Grosso	2021	Soybean	-17.010326			
24	BR-21-Din-138	Mato Grosso	2021	Soybean	-12.733958	Glyphosate	S-metolachlor	Glyphosate
25	BR-21-Din-013	Mato Grosso	2021	Soybean	-17.104991			
26	BR-21-Din-187	Mato Grosso	2021	Soybean	-14.538992	Glyphosate	2,4-D	Glyphosate
27	BR-21-Din-035	Mato Grosso	2021	Soybean	-13.993245	Glyphosate	S-metolachlor	Glyphosate
28	BR-21-Din-021	Mato Grosso	2021	Soybean	-16.832857	Glyphosate	Glyphosate	Clethodim
29	BR-21-Din-179	Mato Grosso	2021	Soybean	-15.364908			

BR-21-Din-060	Mato Grosso	2021	Soybean	-13.155242			
BR-21-Din-090	Mato Grosso	2021	Soybean	-13.812017	Glyphosate	Haloxyfop	
BR-21-Din-132	Mato Grosso	2021	Soybean	-13.06532	Glyphosate	Imazethapyr	Glyphosate
BR-21-Din-175	Mato Grosso	2021	Soybean	-15.458755	Glyphosate	Clethodim	Glyphosate
BR-21-Din-122	Mato Grosso	2021	Soybean	-12.616415			
BR-21-Din-123	Mato Grosso	2021	Soybean	-12.778067			
BR-21-Din-124	Mato Grosso	2021	Soybean	-12.44635			
BR-21-Din-125	Mato Grosso	2021	Soybean	-12.051184			
BR-21-Din-126	Mato Grosso	2021	Soybean	-12.697434			
BR-21-Din-127	Mato Grosso	2021	Soybean	-12.226474			
BR-21-Din-141	Mato Grosso	2021	Soybean	-13.019929			
BR-21-Din-148	Mato Grosso	2021	Soybean	-11.863169	Imazethapyr	Chlorimuron	Glyphosate
BR-21-Din-152	Mato Grosso	2021	Soybean	-11.868124	Glyphosate	2,4-D	Chlorimuron
BR-21-Din-153	Mato Grosso	2021	Soybean	-14.088419			
BR-21-Din-159	Mato Grosso	2021	Soybean	-12.254643			
BR-21-Din-079	Mato Grosso	2021	Soybean	-12.77259			
BR-21-Din-075	Mato Grosso	2021	Soybean	-13.199816			
BR-21-Din-078	Mato Grosso	2021	Soybean	-13.674283			
BR-21-Din-023	Mato Grosso	2021	Soybean	-16.872373			
BR-21-Din-024	Mato Grosso	2021	Soybean	-16.915311	2,4-D	Glyphosate	Chlorimuron
BR-21-Din-073	Mato Grosso	2021	Soybean	-12.581127			
BR-21-Din-074	Mato Grosso	2021	Soybean	-13.473843			
BR-21-Din-064	Mato Grosso	2021	Soybean	-13.493151			
BR-21-Din-055	Mato Grosso	2021	Soybean	-13.73439			
BR-21-Din-088	Mato Grosso	2021	Soybean	-13.409104			
BR-21-Din-121	Mato Grosso	2021	Soybean	-12.635014			
BR-21-Din-129	Mato Grosso	2021	Soybean	-12.630135			
BR-21-Din-089	Mato Grosso	2021	Soybean	-13.30916			
BR-21-Din-077	Mato Grosso	2021	Soybean	-12.5883			
BR-21-Din-131	Mato Grosso	2021	Soybean	-11.970327	Glyphosate	Flumioxazina	Glyphosate
BR-21-Din-160	Mato Grosso	2021	Soybean	-11.827673			

BR-21-Din-158	Mato Grosso	2021	Soybean	-12.503617			
BR-21-Din-128	Mato Grosso	2021	Soybean	-12.83417	Glyphosate	2,4-D	Chlorimuron
BR-21-Din-025	Mato Grosso	2021	Soybean	-16.345974			
BR-21-Din-022	Mato Grosso	2021	Soybean	-14.65118	Chlorimuron	2,4-D	Glyphosate
BR-21-Din-154	Mato Grosso	2021	Soybean	-12.020034	S-metolachlor	Flumioxazina	Glyphosate
BR-21-Din-137	Mato Grosso	2021	Soybean	-13.227811			
BR-21-Din-130	Mato Grosso	2021	Soybean	-12.875366			
BR-21-Din-146	Mato Grosso	2021	Soybean	-12.206939			
BR-21-Din-150	Mato Grosso	2021	Soybean	-14.063788			
BR-21-Din-144	Mato Grosso	2021	Soybean	-14.361704	2,4-D	Glyphosate	Glyphosate
BR-21-Din-039	Mato Grosso	2021	Soybean	-13.652278			
BR-21-Din-038	Mato Grosso	2021	Soybean	-14.076755			
BR-21-Din-082	Mato Grosso	2021	Soybean	-12.661091			
BR-21-Din-084	Mato Grosso	2021	Soybean	-13.779833			
BR-21-Din-087	Mato Grosso	2021	Soybean	-14.082626			
BR-21-Din-151	Mato Grosso	2021	Soybean	-12.235481			
BR-21-Din-037	Mato Grosso	2021	Soybean	-14.066696	Glyphosate	S-metolachlor	
BR-21-Din-033	Mato Grosso	2021	Soybean	-13.868817			
BR-21-Din-285	Minas Gerais	2021		-19.067854			
BR-21-Din-288	Minas Gerais	2021		-18.942667			
BR-21-Din-289	Minas Gerais	2021		-19.036209			
BR-21-Din-291	Minas Gerais	2021		-19.336775			
BR-21-Din-286	Minas Gerais	2021		-18.971354			
BR-21-Din-304	Minas Gerais	2021		-11.92994			
BR-21-Din-294	Minas Gerais	2021		-19.872131			
BR-21-Din-282	Minas Gerais	2021		-20.80399			
BR-21-Din-281	Minas Gerais	2021		-19.623859			
BR-21-Din-297	Minas Gerais	2021		-19.24608			
BR-21-Din-292	Minas Gerais	2021		-18.931199			
BR-21-Din-300	Minas Gerais	2021		-18.9951			
BR-21-Din-293	Minas Gerais	2021		-19.140554			

97	BR-21-Din-287	Minas Gerais	2021		-19.756373			
101	BR-21-Din-301	Minas Gerais	2021		-18.731074			
102	BR-21-Din-299	Minas Gerais	2021		-19.69563			
103	BR-21-Din-298	Minas Gerais	2021		-19.304419			
104	BR-21-Din-295	Minas Gerais	2021		-19.667344			
105	BR-21-Din-283	Minas Gerais	2021		-18.756988			
106	BR-21-Din-002	Mato Grosso do Sul	2021	Soybean	-22.111722	Glyphosate	Haloxyfop	
107	BR-21-Din-421	Mato Grosso do Sul	2021	Corn	-23			
108	BR-21-Din-001	Mato Grosso do Sul	2021	Soybean	-20.405689	Glyphosate	2,4-D	Haloxyfop
109	BR-21-Din-051	Mato Grosso do Sul	2021	Soybean	-22.353837			
110	BR-21-Din-006	Mato Grosso do Sul	2021	Soybean	-18.489468			
111	BR-21-Din-004	Mato Grosso do Sul	2021	Soybean	-22.318326			
112	BR-21-Din-423	Mato Grosso do Sul	2021	Corn	-22.16053			
113	BR-21-Din-008	Mato Grosso do Sul	2021	Soybean	-21.435396	Glyphosate	2,4-D	Clethodim
114	BR-21-Din-003	Mato Grosso do Sul	2021	Soybean	-18.516036	Clethodim	2,4-D	
115	BR-21-Din-053	Mato Grosso do Sul	2021	Soybean	-22.651729			
116	BR-21-Din-257	Bahia	2021	Soybean	-11.655282	Glyphosate	Clethodim	
117	BR-21-Din-255	Bahia	2021	Soybean	-12.34024	Glyphosate	Imazethapyr	Clethodim
118	BR-21-Din-307	Bahia	2021	Corn	-12.351747			
119	BR-21-Din-258	Bahia	2021	Soybean	-11.864937	Glyphosate	Clethodim	
120	BR-21-Din-237	Bahia	2021	Soybean	-11.927766			
121	BR-21-Din-243	Bahia	2021	Soybean	-12.126352			
122	BR-21-Din-244	Bahia	2021		-11.652854			
123	BR-21-Din-246	Bahia	2021	Soybean	-12.590757			
124	BR-21-Din-247	Bahia	2021	Soybean	-12.153097			

125	BR-21-Din-248	Bahia	2021	Soybean	-11.313763			
126	BR-21-Din-256	Bahia	2021	Soybean	-12.462899			
127	BR-21-Din-260	Bahia	2021	Soybean	-11.913335			
128	BR-21-Din-264	Bahia	2021		-13.194513			
129	BR-21-Din-265	Bahia	2021		-13.079499			
130	BR-21-Din-268	Bahia	2021	Soybean	-12.800007			
131	BR-21-Din-250	Bahia	2021	Soybean	-12.36687			
132	BR-21-Din-251	Bahia	2021	Soybean	-12.226976			
133	BR-21-Din-252	Bahia	2021	Soybean	-11.70877			
134	BR-21-Din-253	Bahia	2021	Soybean	-11.507872			
135	BR-21-Din-254	Bahia	2021	Soybean	-12.126352			
136	BR-21-Din-261	Bahia	2021	Soybean	-13.807359			
137	BR-21-Din-262	Bahia	2021	Soybean	-13.859497			
138	BR-21-Din-263	Bahia	2021	Soybean	-13.676489			
139	BR-21-Din-270	Bahia	2021	Soybean	-12.727537			
140	BR-21-Din-165	Goiás	2021		-17.797813			
141	BR-21-Din-169	Goiás	2021	Soybean	-17.691461	Glyphosate	Clethodim	Glyphosate
142	BR-21-Din-220	Goiás	2021	Soybean	-17.425761	Glyphosate	S-metolachlor	Glyphosate
143	BR-21-Din-167	Goiás	2021	Soybean	-17.670061			
144	BR-21-Din-168	Goiás	2021		-17.792562			
145	BR-21-Din-223	Goiás	2021	Soybean	-17.148551			
146	BR-21-Din-225	Goiás	2021	Soybean	-17.806397			
147	BR-21-Din-226	Goiás	2021		-17.919299			
148	BR-21-Din-227	Goiás	2021	Soybean	-17.035684			
149	BR-21-Din-233	Goiás	2021	Soybean	-17.108197			
150	BR-21-Din-234	Goiás	2021	Soybean	-15.617146	Glyphosate	2,4-D	Carfentrazona
151	BR-21-Din-235	Goiás	2021	Soybean	-17.492984			
152	BR-21-Din-236	Goiás	2021	Soybean	-17.024766			
153	BR-21-Din-219	Goiás	2021		-15.789144			
154	BR-21-Din-221	Goiás	2021		-17.090348			
155	BR-21-Din-222	Goiás	2021	Soybean	-17.650682			

BR-21-Din-228	Goiás	2021		-18.171877			
BR-21-Din-229	Goiás	2021		-17.660136			
BR-21-Din-232	Goiás	2021		-17.177725			
BR-21-Din-230	Goiás	2021	Soybean	-17.345809			
BR-21-Din-417	Paraná	2021	Corn	-24.929087			
BR-21-Din-042	Paraná	2021	Soybean	-24.118358	Glyphosate	Paraquat	Haloxyfop
BR-21-Din-047	Paraná	2021	Soybean	-24.128862	Haloxyfop	Glyphosate	
BR-21-Din-041	Paraná	2021	Soybean	-25.134125			
BR-21-Din-419	Paraná	2021	Corn	-24.921053			
BR-21-Din-044	Paraná	2021	Soybean	-24.646131	Glyphosate	Glyphosate	Clethodim
BR-21-Din-048	Paraná	2021	Soybean	-24.112569			
BR-21-Din-097	Paraná	2021	Soybean	-24.534918	Clethodim	Glyphosate	
BR-21-Din-197	Paraná	2021	Soybean	-23.144235			
BR-21-Din-198	Paraná	2021	Soybean	-23.234739			
BR-21-Din-199	Paraná	2021	Soybean	-23.18473			
BR-21-Din-200	Paraná	2021	Soybean	-23.25644			
BR-21-Din-201	Paraná	2021	Soybean	-23.152238			
BR-21-Din-203	Paraná	2021	Soybean	-23.222232			
BR-21-Din-206	Paraná	2021	Soybean	-23.130442			
BR-21-Din-115	Paraná	2021	Soybean	-25.01236			
BR-21-Din-099	Paraná	2021		-24.000413			
BR-21-Din-110	Paraná	2021	Soybean	-23.492098			
BR-21-Din-107	Paraná	2021	Soybean	-23.841235			
BR-21-Din-118	Paraná	2021	Soybean	-23.471419			
BR-21-Din-195	Paraná	2021	Soybean	-25.530857			
BR-21-Din-196	Paraná	2021	Soybean	-23.379135			
BR-21-Din-193	Paraná	2021	Soybean	-23.100551			
BR-21-Din-192	Paraná	2021	Soybean	-24.98059			
BR-21-Din-191	Paraná	2021	Soybean	-25.379135			
BR-21-Din-207	Paraná	2021	Soybean	-23.047561	Glyphosate	Glyphosate	
BR-21-Din-116	Paraná	2021	Soybean	-24.50212			

BR-21-Din-100	Paraná	2021		-25.43785			
BR-21-Din-112	Paraná	2021	Soybean	-23.830969			
BR-21-Din-095	Paraná	2021	Soybean	-24.466581			
BR-21-Din-205	Paraná	2021	Soybean	-23.158959			
BR-21-Din-202	Paraná	2021	Soybean	-22.995947			
BR-21-Din-204	Paraná	2021	Soybean	-23.157074			
BR-21-Din-092	Paraná	2021	Soybean	-24.627952			
BR-21-Din-091	Paraná	2021	Soybean	-24.915613			
BR-21-Din-212	Rio Grande do Sul	2021	Soybean	-27.905476			
BR-21-Din-240	Rio Grande do Sul	2021	Soybean	-28.012025			
BR-21-Din-241	Rio Grande do Sul	2021	Soybean	-28.176374			
BR-21-Din-242	Rio Grande do Sul	2021	Soybean	-28.220694			
BR-21-Din-218	Rio Grande do Sul	2021	Soybean	-28.123066			
BR-21-Din-217	Rio Grande do Sul	2021	Soybean	-28.220091			
BR-21-Din-216	Rio Grande do Sul	2021	Soybean	-28.286886			
BR-21-Din-215	Rio Grande do Sul	2021	Soybean	-27.9067			
BR-21-Din-214	Rio Grande do Sul	2021	Soybean	-28.327221			
BR-21-Din-209	Rio Grande do Sul	2021	Soybean	-27.807909			
BR-21-Din-213	Rio Grande do Sul	2021	Soybean	-28.54547			
BR-21-Din-276	Rio Grande do Sul	2021	Soybean	-28.133842			
BR-21-Din-277	Rio Grande do Sul	2021	Soybean	-27.749641			
BR-21-Din-279	Rio Grande do Sul	2021	Soybean	-27.918851			
BR-21-Din-280	Rio Grande do Sul	2021	Soybean	-28.392055			
BR-21-Din-272	Rio Grande do Sul	2021	Soybean	-27.7892			
BR-21-Din-273	Rio Grande do Sul	2021	Soybean	-27.724563			
BR-21-Din-275	Rio Grande do Sul	2021	Soybean	-27.76591			
BR-21-Din-211	Santa Catariana	2021	Soybean	-26.311266	Glyphosate	2,4-D	Glyphosate
BR-21-Din-238	Tocantins	2021		-10.758534			

Table S2 Marker distribution of SNPs and physical genome covered by them on the reference genome of *D. insularis*

Scaffold/chromosome	Number of SNPs	Physical genome covered by SNPs (bp)/Total genome covered (Mb)
S01	737	184921-93914502/93.7
S02	842	976332-82191662/81.2
S03	663	535966-76516551/75.9
S04	654	449414-74436434/73.9
S05	557	87175-69157502/69.0
S06	344	1225484-64452436/63.2
S07	487	327434-59944807/59.6
S08	480	103462-55825410/55.7
S09	474	1117611-53028503/51.9

Table S3 Candidate gene hits identified for the top 15 outlier SNPs

Chromosome/scaffold	Fst	Candidate gene hit	Molecular function	Biological pathways
Ch 1	0.0428	AT1G27930	DUF579: glucuronoxylan 4-O-methyltransferase-like protein	Growth and development
Ch 1	0.0555	AT1G69910	Protein kinase superfamily protein	Stress pathway
Ch 2	0.0426	AT3G46770	P2/B3-like transcriptional factor family	Stress pathway
Ch 2	0.0451	AT1G73200	DUF2404	Growth and development
Ch 2	0.0427	AT2G14260	PIP description:proline iminopeptidase	Stress pathway
Ch 2	0.0422	AT2G14260	PIP description:proline iminopeptidase	Stress pathway
Ch 2	0.0499	AT2G14260	PIP description:proline iminopeptidase	Stress pathway
Ch 3	0.048	AT1G13330	gene_symbol:AHP2 description:Tat-binding protein 1(Tbp-1)-interacting protein (TBPIP)	
Ch 4	0.0433	AT3G23730	XTH16 description:xyloglucan endotransglucosylase/hydrolase 16	Growth and development
Ch 5	0.0518			
Ch 5	0.0518			
Ch 7	0.0479			
Ch 8	0.0417	AT5G40250	RING/U-box superfamily protein	
Ch 9	0.0443	AT1G63100	GRAS family transcription factor	Stress pathway
Ch 9	0.0458	AT1G63100	GRAS family transcription factor	Stress pathway

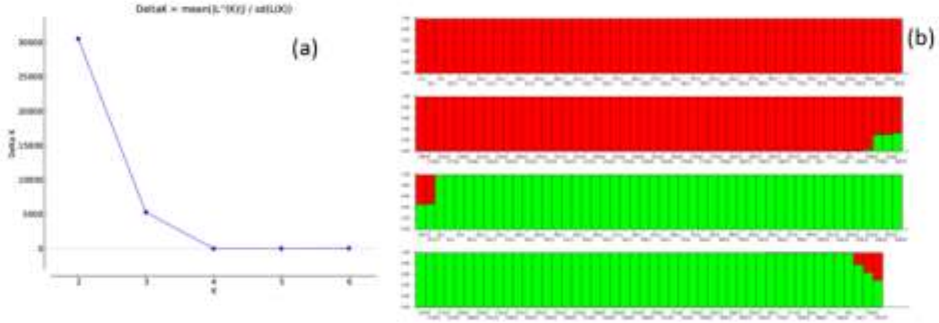

Given Pop	Inferred Clusters 1	2	Number of Individuals
1 (MT)	0.597	0.403	74
2 (MS)	0.600	0.400	10
3 (**MG**)	**0.125**	**0.875**	24
4 (BA)	0.736	0.264	19
5 (GO)	0.350	0.650	20
6 (PR)	0.507	0.493	33
7 (RS)	0.568	0.432	18

Fig. S1 Delta K vs K graph showing the best K as K = 2 (a) and bar plot showing two subpopulations revealed at the best K = 2 (b). Each vertical bar represents an individual and the numbers below bars within brackets represent the population number they belong to. The cluster membership of populations in the two clusters is shown on the right. If the cluster membership >0.80 for a cluster, the population belongs to that cluster.

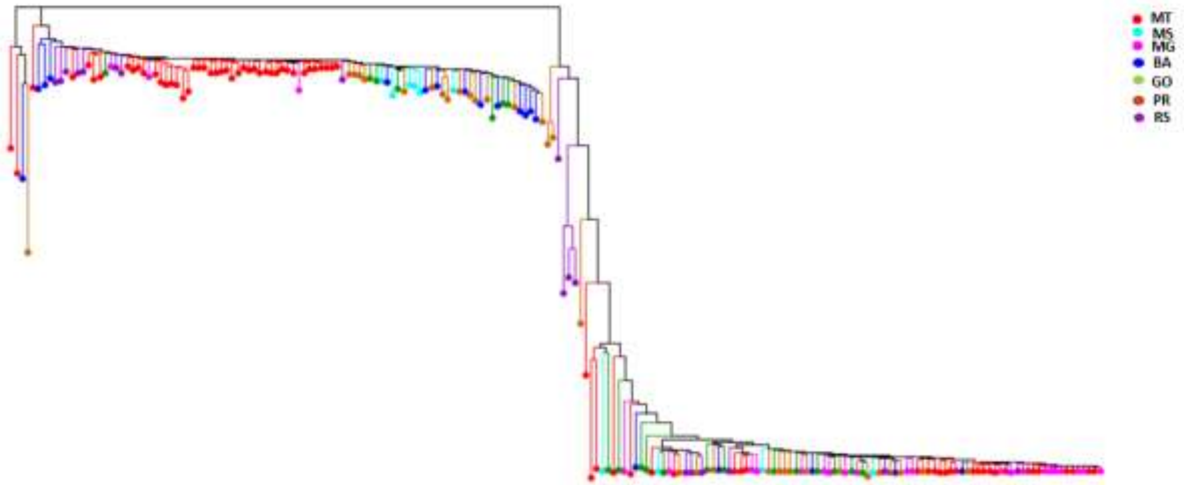

Fig. S2 Weighted NJ tree of *D. insularis* populations based on 5,238 SNPs

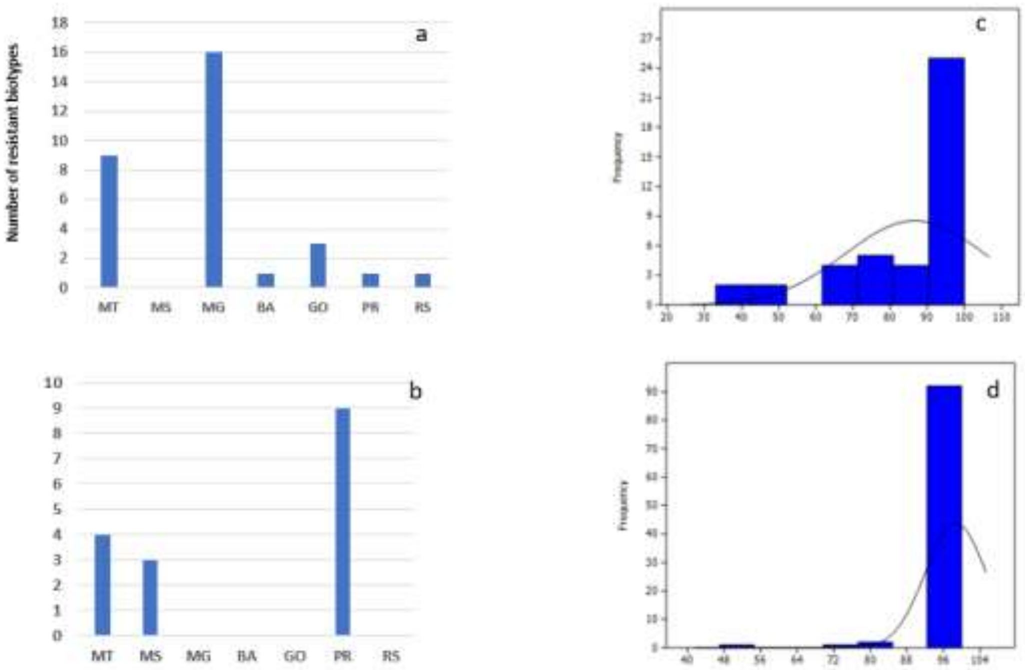

Fig. S3 Number of resistant biotypes observed at a haloxyfop dose of 7.8g (a), clethodim dose of 27.0g (b) and histograms showing normal and near-normal distribution of resistance score for haloxyfop (c) and clethodim (d), respectively.
